# Adverse drug reaction classification by health professionals: appropriate discrimination between allergy and intolerance?

**DOI:** 10.1186/s13601-019-0259-6

**Published:** 2019-03-19

**Authors:** Sepehr Shakib, Gillian E. Caughey, Jie Shen Fok, William B. Smith

**Affiliations:** 1 Department of Clinical Pharmacology, Royal Adelaide Hospital, Adelaide, SA Australia; 2 Discipline of Pharmacology, Adelaide Medical School, Faculty of Health and Medical Sciences, University of Adelaide, Adelaide, SA Australia; 3 School of Pharmacy and Medical Sciences, University of South Australia, Adelaide, SA Australia; 4 Clinical Immunology and Allergy, Royal Adelaide Hospital, North Terrace, Adelaide, SA Australia

**Keywords:** Adverse drug reaction, Allergy, Health services research

## Abstract

**Background:**

The correct classification of an adverse drug reaction (ADR) as allergy (immunological) or intolerance (non-immunological) has important clinical implications. The aim of this study was to examine the ability of health professionals to discriminate between allergy and intolerance, classify the severity of the ADR and degree of contraindication.

**Methods:**

Health professionals were presented ten ‘real-life’ ADR scenarios using an online questionnaire and asked to: categorise the reaction as allergy or intolerance, rate the severity of the reaction and judge the level of contraindication of the causative drug. The number and proportion of responses were calculated for each of the cases presented and associations between classification of reaction type, severity and level of contraindication were examined.

**Results:**

A total of 394 responses were received. Overall 59.0% (SD 28.9) correctly categorised the cases, 60.8% (SD 16.8) classified the severity correct, and less than half (44.7%, SD 28.6) correctly identified the level of contraindication. The proportion of health professionals correctly answering the type, severity and level of contraindication for the allergy case was significantly higher (*p* < 0.0001) by comparison to the intolerance cases (type: 56.6% ± 33.1; severity: 57.3 ± 11.9; level of contraindication: 38.5 ± 19.9).

**Conclusions:**

Health professionals have suboptimal understanding of classification of ADRs. Strategies are required to strictly avoid re-exposure of patients to drugs which carry an increased risk of inducing a dangerous reaction, whilst minimising the avoidance of drugs which are of minimal risk or allowing the use of low-risk drugs where the benefits may be significant.

## Introduction

In Australia, medication-related incidents are estimated to account for 2–3% of all hospital admissions [1]. Using ICD-10 codes, adverse drug reactions (ADRs) account for at least 1.3% of all admissions, whilst an ADR occurs on admission or during a hospital stay in 2.7–3.3% of patients [2, 3]. ADRs range from minor common side effects to potentially life threatening, and are an important public health problem [4]. The traditional pharmacological classification of ADRs includes two major subtypes; type A which are dose-dependent and predictable (non-immunological, commonly termed intolerance), and type B (immunological-allergic) reactions which are unpredictable and not dose-dependent [5]. The majority (≥ 85%) of ADRs are type A (non-immunological), resulting from the pharmacological activity of the drug [4, 6].

The correct classification of an ADR as allergy or intolerance has important clinical implications, especially with regard to future exposure or avoidance of the drug. In the case of allergy, avoidance of the same drug and structurally-related drugs in any dose is required, whereas for intolerance, avoidance of the same drug and pharmacologically-related drugs is recommended, but the risk can also be mitigated by altering the dose or formulation, or by the administration of other medications [4]. Allergy is commonly assumed to be more serious, but intolerance may also be life-threatening, therefore reaction severity might be a more important parameter than mechanistic classification.

Mislabelling of a drug allergy in patient records may result in unnecessary avoidance of an effective drug, resulting in prescribing of a second-line therapy which may be less effective or more expensive, and potentially may lead to higher rates of adverse effects and in the case of penicillins, multi-drug resistant organisms [7, 8]. Furthermore, mislabelling has been shown to be associated with poorer health outcomes for patients [9, 10].

Electronic health records (EHR) have been implemented in many large healthcare organisations world-wide to improve the quality and efficiency of healthcare but have also been associated with poor interoperability and functionality [11, 12]. EHRs provide an opportunity to improve the quality and accuracy of ADR reporting but are highly dependent on the level of documentation by clinicians from accurate patient medical histories or previous records. Further, if the ADR is poorly documented in EHRs, an inaccurate label may persist, potentially affecting future appropriate prescribing decisions. Indeed, our recent study demonstrated over 20% of reported ADRs did not contain a reaction description and categorisation of allergy and intolerance was inconsistent when penicillin ADRs were documented in a hospital EHR for over 5000 patients [13]. It is not known whether this inconsistent documentation is due to poor underlying understanding of ADR types, or difficulties in documentation in the electronic system. The aim of this study was to examine the ability of health professionals to discriminate between allergy and intolerance, in standardised clinical scenarios, and to classify the severity of the ADR and degree of drug contraindication.

## Methods

### Ethics approval

The study was approved by the Royal Adelaide Hospital Human Research Ethics Committee (Approval No. 130617).

### Clinical questionnaire

Ten ‘real-life’ ADR scenarios with a range of mechanisms and severity, including two control “obvious” questions were developed (Appendix). One control was for severe anaphylaxis where the drug should not be administered again (Scenario 8) and the other for mild intolerance, where the drug could be used in the future with caution (Scenario 2). There was one allergy case and seven intolerance cases, based on common medication classes and ADRs, where in the experience of the authors, confusion often arose regarding the correct allergy or intolerance attribution [3, 6].

In each case participants were asked:

- To categorise the reaction as allergy or intolerance
- To rate the severity of the reaction (mild, moderate, or severe), and
- To judge the level of contraindication of the causative drug (absolutely contraindicated, relatively contraindicated, or use with caution).

The questionnaire was made available online through auditmaker.net (an online tool for clinical audit) as well as paper-based if required. Data collected from paper forms were manually entered into auditmaker.net. The severity of the reaction was classified according to the Internationally regognized Common Terminology Criteria for Adverse Events (CTCAE) version 4.0 for the classification of Mild (Grade 1: asymptomatic or mild symptoms; clinical or diagnostic observations only; intervention not indicated); Moderate (Grade 2: minimal symptoms; local or non-invasive intervention indicated; limiting age-appropriate instrumental activities of daily living) or Severe (Grade 3: medically significant but not immediately life-threatening; hospitalization or prolongation of hospitalization indicated; disabling; limiting self-care activities of daily living) [14]. The level of severity and contraindication was determined by an expert panel (clinical immunologist and clinical pharmacologist) together with current evidence from a national (Australian) evidence-based medicine reference [15].

### Participants

Hospital-based health-care workers (HCW) were invited to complete the questionnaire through invitation by email through the investigators’ professional networks, as well as through teaching sessions, continuing education meetings, and other hospital meetings.

### Statistical analysis

The number and proportion of responses by HCW were calculated for each of the cases presented. The association between classification of reaction type, severity and level of contraindication by health professional was examined for three representative cases. Statistical analysis was undertaken using SPSS with Chi Squared tests used to compare significant differences between health professionals and correct responses. A *p* value of < 0.05 was regarded as significant.

## Results

A total of 394 responses from HCW and medical students were received. 160 (40.6%) were from medical practitioners, 50 (12.7%) from nurses, 96 (24.4%) from pharmacists and 88 (22.3%) from medical students. Medical practitioners included a range of specialties with varying levels of experience from interns (27.5%), basic and advanced internal medicine trainees (56.3%), to clinical specialists (16.2%) from a range of disciplines including immunology (6.9%), clinical pharmacology (4.4%) and other specialties (88.7%).

Shown in Table 1 are the responses for the ten clinical scenarios. Over 97% of respondents correctly identified the ‘control’ case of allergy (penicillin-induced anaphylaxis) with the highest correct response from medical practitioners (99.4%). Overall 96.7% of respondents identified the severity correctly and 97.7% correctly reported it to be absolutely contraindicated. For the second allergy case presented, of a hypersensitivity reaction to carbamazepine manifesting in Stevens–Johnson syndrome (SJS), overall 76% of health professionals correctly identified this as allergy. Only 52% of nurses correctly identified this, by contrast to pharmacists where 83.3% provided the correct answer. Almost 90% of participants documented the severity and level of contraindication correctly but again only 52% of nurses correctly identified these for this reaction.

**Table 1:** Health professional assessment of clinical scenarios for type and severity of reaction and level contraindication

Health profession	Type of reaction N (%)		Severity of reaction N (%)			Level of contraindication N (%)		
	Allergy	Intolerance	Mild	Moderate	Severe	Absolutely	Relatively	Use with caution
Allergy								
Control—Augmentin, anaphylaxis (severe, absolutely contraindicated)								
Medical (n = 160)	*159 (99.4)*	0	1 (0.6)	2 (1.3)	*156 (97.5)*	*158 (98.8)*	0	1 (0.6)
Nurse (n = 50)	*49 (98.0)*	0	1 (2.0)	2 (4.0)	*46 (92.0)*	*46 (92.0)*	3 (6.0)	0
Pharmacist (n = 96)	*94 (97.9)*	2 (2.1)	0	3 (3.1)	*93 (96.9)*	*94 (97.9)*	1 (1.0)	1 (1.0)
Medical student (n = 88)	*86 (97.7)*	1 (1.1)	0	1 (1.1)	*86 (97.7)*	*87 (98.9)*	0	0
Overall (n = 394)	*388 (98.5)*	3 (0.76)	2 (0.51)	8 (2.0)	*381 (96.7)*	*385 (97.7)*	4 (1.0)	2 (0.51)
Carbamazepine, Stevens–Johnson syndrome (severe, absolutely contraindicated)								
Medical (n = 160)	*128 (80.0)*	31 (19.4)	0	17 (10.6)	*142 (88.8)*	*148 (92.5)*	12 (7.5)	0
Nurse (n = 50)	*26 (52.0)*	24 (48.0)	0	12 (24.0)	*26 (52.0)*	*26 (52.0)*	20 (40.0)	4 (8.0)
Pharmacist (n = 96)	*80 (83.3)*	16 (16.7)	0	3 (3.1)	*93 (96.9)*	*93 (96.9)*	3 (3.1)	0
Medical student (n = 88)	*67 (76.1)*	20 (22.7)	0	5 (5.6)	*83 (89.8)*	*79 (89.8)*	9 (10.2)	0
Overall (n = 394)	*301 (76.4)*	91 (23.1)	0	37 (9.4)	*344 (87.3)*	*346 (87.8)*	44 (11.2)	4 (1.0)
Intolerance								
Control—PPI, headache (mild, use with caution)								
Medical (n = 160)	1 (0.6)	*157 (98.1)*	*157 (98.1)*	3 (1.9)	0	0	47 (29.4)	*113 (70.6)*
Nurse (n = 50)	6 (12.0)	*43 (86.0)*	*45 (90.0)*	4 (8.0)	0	1 (2.0)	17 (34.0)	*30 (60.0)*
Pharmacist (n = 96)	1 (1.0)	*94 (98.0)*	*91 (94.8)*	5 (5.2)	0	0	33 (34.4)	*62 (65.6)*
Medical student (n = 88)	0	*88 (100)*	*83 (94.3)*	5 (5.7)	0	0	26 (11.4)	*62 (70.5)*
Overall (n = 394)	8 (2.0)	*382 (96.9)*	*376 (95.4)*	(4.8)	0	1 (0.25)	123 (31.2)	*267 (67.8)*
Statin, rhabdomyolysis (severe, use with caution)								
Medical (n = 160)	32 (20.0)	*124 (77.5)*	0	45 (28.1)	*115 (71.9)*	92 (57.5)	59 (36.9)	*9 (5.6)*
Nurse (n = 50)	10 (20.0)	*38 (76.0)*	5 (10.0)	18 (36.0)	*25 (50.0)*	21 (42.0)	18 (36.0)	*10 (20.0)*
Pharmacist (n = 96)	15 (15.6)	*80 (83.3)*	0	19 (19.8)	*77 (80.2)*	49 (51.0)	41 (42.7)	*6 (6.3)*
Medical student (n = 88)	13 (14.8)	*73 (82.9)*	0	11 (12.5)	*76 (86.4)*	53 (60.2)	32 (36.4)	*2 (2.3)*
Overall (n = 394)	70 (17.8)	*315 (79.9)*	5 (1.3)	93 (23.6)	*293 (74.4)*	215 (54.6)	150 (38.1)	*27 (6.9)*
Azathioprine, myelosuppression, hepatitis (moderate, absolutely contraindicated)								
Medical (n = 160)	24 (15.0)	*130 (81.3)*	0	*65 (40.6)*	76 (47.5)	*49 (30.6)*	77 (48.1)	33 (20.6)
Nurse (n = 50)	15 (30.0)	*34 (68.0)*	0	*31 (62.0)*	19 (38.0)	*17 (34.0)*	22 (44.0)	11 (22.0)
Pharmacist (n = 96)	17 (17.7)	*79 (82.3)*	1 (1.0)	*54 (56.3)*	40 (41.7)	*20 (20.8)*	54 (56.3)	22 (22.9)
Medical student (n = 88)	13 (14.8)	*74 (84.1)*	1 (1.1)	*50 (56.8)*	36 (40.9)	*27 (30.7)*	54 (61.4)	6 (6.8)
Overall (n = 394)	69 (17.5)	*317 (80.4)*	2 (0.5)	*200 (50.7)*	171 (43.4)	*113 (28.7)*	207 (52.5)	72 (18.3)
ACE, angioedema (moderate, absolutely contraindicated)								
Medical (n = 160)	109 (68.1)	*48 (30.0)*	14 (8.8)	*82 (51.3)*	63 (39.4)	*110 (68.8)*	45 (28.1)	4 (2.5)
Nurse (n = 50)	38 (76.0)	*11 (22.0)*	4 (8.0)	*30 (60.0)*	16 (32.0)	*29 (58.0)*	18 (36.0)	3 (6.0)
Pharmacist (n = 96)	73 (76.0)	*23 (23.9)*	1 (1.0)	*30 (31.3)*	65 (67.7)	*87 (90.6)*	9 (9.4)	0
Medical student (n = 88)	74 (84.1)	*14 (15.9)*	9 (10.2)	*51 (58.0)*	28 (31.8)	*36 (40.9)*	46 (52.3)	6 (6.8)
Overall (n = 394)	294 (74.6)	*96 (24.4)*	28 (7.1)	*193 (49.0)*	172 (43.7)	*262 (66.5)*	118 (29.9)	13 (3.3)
NSAIDs, hives (moderate, relatively contraindicated)								
Medical (n = 160)	144 (90.0)	*14 (8.8)*	23 (14.4)	*121 (75.6)*	14 (8.8)	67 (41.9)	*76 (47.5)*	15 (9.4)
Nurse (n = 50)	39 (78.0)	*9 (18.0)*	17 (34.0)	*31 (62.0)*	2 (4.0)	14 (28.0)	*6 (12.0)*	30 (60.0)
Pharmacist (n = 96)	91 (94.8)	*5 (5.2)*	19 (19.8)	*64 (66.7)*	13 (13.5)	42 (43.8)	*37 (38.5)*	16 (16.7)
Medical student (n = 88)	85 (96.6)	*3 (3.4)*	10 (11.4)	*67 (76.1)*	10 (11.4)	31 (35.2)	*49 (55.7)*	8 (9.1)
Overall (n = 394)	359 (91.1)	*31 (7.9)*	69 (17.5)	*283 (71.8)*	39 (9.9)	154 (39.1)	*168 (42.6)*	69 (17.5)
Beta-blocker, shortness of breath (moderate, relatively contraindicated)								
Medical (n = 160)	18 (11.2)	*139 (86.9)*	37 (23.1)	*112 (70.0)*	8 (5.0)	28 (17.5)	*109 (68.1)*	19 (11.9)
Nurse (n = 50)	22 (44.0)	*26 (52.0)*	15 (30.0)	*28 (56.0)*	5 (10.0)	14 (28.0)	*20 (40.0)*	14 (28.0)
Pharmacist (n = 96)	4 (4.2)	*91 (94.8)*	28 (29.2)	*61 (63.5)*	7 (7.3)	13 (13.5)	*57 (59.4)*	25 (26.0)
Medical student (n = 88)	12 (13.6)	*76 (86.4)*	31 (35.2)	*53 (60.2)*	4 (4.5)	11 (12.5)	*61 (69.3)*	16 (18.2)
Overall (n = 394)	56 (14.2)	*332 (84.3)*	111 (28.2)	*254 (64.5)*	24 (6.1)	66 (16.8)	*247 (62.7)*	74 (18.8)
Morphine, hives (mild, use with caution)								
Medical (n = 160)	114 (71.3)	*44 (27.5)*	*93 (58.1)*	64 (40.0)	2 (1.25)	22 (13.8)	104 (65.0)	*33 (20.6)*
Nurse (n = 50)	28 (56.0)	*20 (40.0)*	*21 (42.0)*	28 (56.0)	0	7 (14.0)	26 (52.0)	*16 (32.0)*
Pharmacist (n = 96)	66 (68.8)	*30 (31.3)*	*35 (36.5)*	58 (60.4)	3 (3.1)	11 (11.5)	63 (65.6)	*22 (22.9)*
Medical student (n = 88)	82 (93.2)	*5 (5.7)*	*38 (43.2)*	48 (54.5)	1 (1.1)	10 (11.4)	63 (71.6)	*14 (15.9)*
Overall (n = 394)	290 (73.6)	*99 (25.1)*	*187 (47.5)*	198 (50.3)	6 (1.5)	50 (12.7)	256 (65.0)	*85 (21.6)*
Erythromycin, GI complaint (moderate, relatively contraindicated)								
Medical (n = 160)	5 (3.1)	*151 (94.4)*	101 (63.1)	*58 (36.3)*	0	2 (1.3)	*61 (38.1)*	95 (59.4)
Nurse (n = 50)	5 (10.0)	*43 (86.0)*	17 (34.0)	*31 (62.0)*	1 (2.0)	4 (8.0)	*24 (48.0)*	21 (42.0)
Pharmacist (n = 96)	3 (3.1)	*93 (96.9)*	69 (71.9)	*23 (24.0)*	1 (1.0)	1 (1.0)	*26 (27.1)*	66 (68.8)
Medical student (n = 88)	4 (4.5)	*83 (94.3)*	38 (43.2)	*49 (55.7)*	0	0	*49 (55.7)*	38 (43.2)
Overall (n = 394)	17 (4.3)	*370 (93.9)*	225 (57.1)	*161 (40.9)*	2 (0.5)	7 (1.8)	*160 (40.6)*	220 (55.8)

Cells may not add up to 100% due to missing data

Those columns highlighted in italics are the correct answers

The level of accuracy for the type and severity of the intolerance ‘control’ case [proton pump inhibitor (PPI)-induced headache] was 96.9% and 95.4% for all health professionals, respectively (Table 1). However, only 67.8% of respondents correctly identified the level of contraindication as use with caution; 31.2% reported it to be relatively contraindicated. Overall for the seven cases of intolerance there was a wide variation in the level of correct responses. The case of erythromycin induced gastrointestinal (GI) complaint had the highest proportion of correct responses for type of reaction at 93.9% overall (ranging from 86.0% for nurses to 96.9% for pharmacists). The least correct classification of intolerance was observed for non-steroidal anti-inflammatory (NSAID)-induced hives where only 7.9% of health professionals reported it correctly. Statin-induced rhabdomyolysis had the highest proportion of correct responses for classification of severity (severe) by participants (74.4%) with 86.4% of medical students correctly classifying this by comparison to only half of nurses. Overall correct classification of the level of contraindication for intolerances was low ranging from 6.9% for statin induced rhabdomyolysis to 66.5% for angiotensin-converting-enzyme (ACE) induced angioedema where it was absolutely contraindicated. Of concern, only 28.7% of HCW stated that azathioprine would be absolutely contraindicated following azathioprine-induced myelosuppression and hepatitis.

**Fig. 1:**
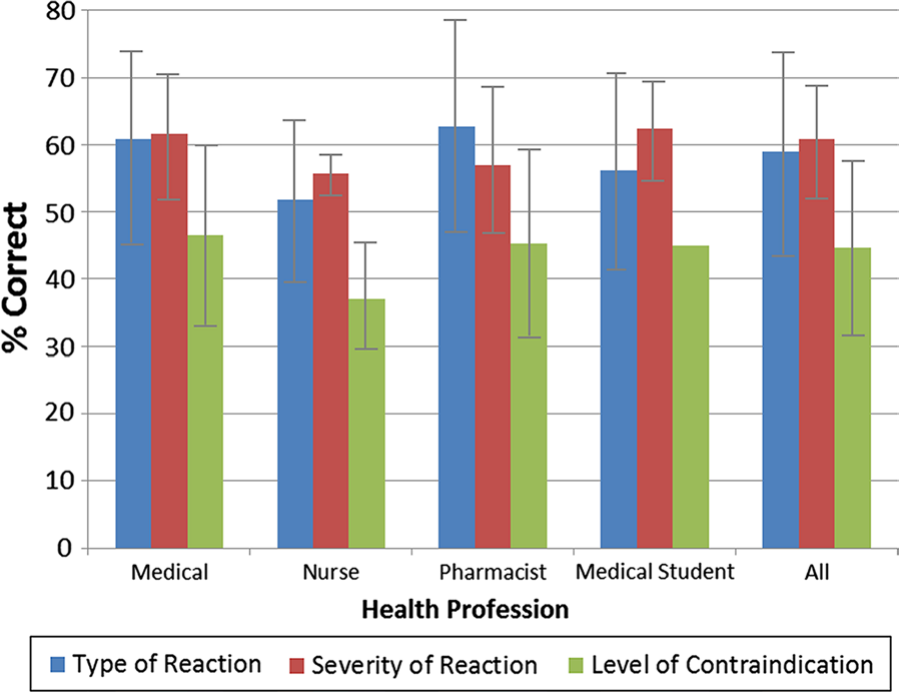
Proportion of correct responses to clinical scenarios for type, severity of reaction and level contraindication by health profession

Figure 1 depicts the overall proportion of correct responses to the clinical scenarios (excluding the control case of allergy and the control case for intolerance) for type, severity of reaction and level contraindication by health profession. There were no significant differences between health professionals for correct classification of type, severity or level of contraindication. Overall, approximately 60% of health professionals correctly identified the type and severity of reactions and 45% correctly identified the level of contraindication. The proportion of health professionals correctly answering the type, severity and level of contraindication for the allergy case was significantly higher (*p* < 0.0001) by comparison to the intolerance cases (type: 56.6% ± 33.1; severity: 57.3 ± 11.9; level of contraindication: 38.5 ± 19.9).

We next examined the level of association between classification of reaction type, severity and level of contraindication between health professionals for three of the clinical cases representing a range of reaction types, severity and contraindication, as shown in Table 2. Overall, for all health professionals, correct classification of the severity of both the ADR and level of contraindication was significantly higher than correct classification of the type of ADR. For the allergy case approximately 70% of health professionals were able to identify the correct type and severity or type and level of contraindication, and over 80% were able to describe the correct severity and level of contraindication. By comparison for the intolerance cases, correct reporting occurred by 25% or less for all health professionals with correct classification of severity and level of contraindication reported significantly more than type of reaction (Table 2).

**Table 2:** Association between classification of reaction type, severity and level of contraindication by health professional

Health profession	Correct classification of reaction type and severity, N (%)	Correct classification of reaction type and contraindication, N (%)		Correct classification of severity and contraindication, N (%)
Carbamazepine, Stevens–Johnson syndrome (severe, absolutely contraindicated)				
Medical (n = 160)	118 (73.8)	122 (76.3)		138 (86.3)*^,^**
Nurse (n = 50)	15 (30.0)	19 (38.0)		18 (36.0)
Pharmacist (n = 96)	77 (80.2)	78 (81.3)		90 (93.8)*^,^**
Medical student (n = 88)	64 (72.7)	63 (71.6)		75 (85.2)*^,^**
Overall (n = 394)	274 (69.5)	282 (71.6)		321 (81.5)*^,^**
ACE, angioedema (moderate, absolutely contraindicated)				
Medical (n = 160)	24 (15.0)	32 (22.5)		48 (30.0)*^,^**
Nurse (n = 50)	7 (14.0)	3 (6.0)		16 (32.0)*^,^**
Pharmacist (n = 96)	7 (7.3)	19 (19.8)*		22 (22.9)*
Medical student (n = 88)	9 (10.2)	2 (2.3)*		13 (14.8)**
Overall (n = 394)	47 (11.9)	56 (14.2)		99 (25.1)*^,^**
Morphine, hives (mild, use with caution)				
Medical (n = 160)	36 (22.5)	21 (13.1)*		31 (19.4)**
Nurse (n = 50)	11 (22.0)	10 (20.0)		13 (26.0)
Pharmacist (n = 96)	12 (13.5)	11 (12.5)		17 (17.7)
Medical student (n = 88)	3 (3.4)	3 (3.4)		11 (12.5)*^,^**
Overall (n = 394)	62 (15.7)	45 (11.4)		72 (18.3)**

*p < 0.05 by comparison to ‘correct classification of reaction type and severity’

**p < 0.05 by comparison to ‘correct classification of reaction type and contraindication’

## Discussion

The accurate reporting and documentation of ADRs is an integral component of pharmacovigilance and patient safety, with major implications for future use or avoidance of the drug. Whilst some medications should be strictly avoided or used only in the context of desensitisation protocols, unnecessary avoidance of medications based on inaccurate information within the EHR may place patients at increased risk of poor health outcomes [9, 10]. ADR information is entered into the EHR by a wide range of HCW with different educational backgrounds, and often relies on interpretation of the patient history, which may itself be unreliable. The results of this study have shown that given a standardised clinical scenario, HCW show a poor understanding of ADRs in terms of classification as allergy or intolerance, severity, and level of contraindication of the drug.

Although almost three quarters of HCW were able to correctly identify an allergic ADR and its level of severity and contraindication, up to 40% of clinicians would re-expose a patient with a diagnosis of carbamazepine-induced SJS to carbamazepine, including 7.5% of all medical staff. This highlights the importance of allergy checking alerts to fill in gaps in clinician awareness. The correct categorisation of intolerance reactions was lower, down to 8% in some scenarios. This may result from the tendency to choose allergy as the default option for all ADRs. This misperception is not helped by many EHRs which use an icon entitled “Allergies” to enter any adverse reaction. Also in some cases, the clinical features of the pharmacological reaction, for example opiate urticaria, NSAID urticaria and ACE-inhibitor angioedema resemble allergic reactions [16].

Overriding of prescribing alerts has been shown to be common, presumably at least in part because mild or trivial reactions may also generate alerts [17, 18]. There is evidence that a number of strategies including tiered alerts can help reduce alert fatigue [19]. However, building in tiered alerts such as different levels of alerts for allergies and intolerances or for different levels of severity or contraindication into EHRs is difficult [19]. Differential alerting based on the allergy or intolerance categorisation would not be appropriate since non-immunological reactions can also be dangerous. Indeed, two of our eight intolerance scenarios were associated with absolute drug contraindication. Tiering of alerts could be based on reaction severity but even this might not predict future risk. Ideally tiering should reflect risk in terms of both likelihood and severity of any reaction that could occur.

Since categorisation of reaction type is demonstrably poor, this raises the question as to whether assessment of severity may be a more important parameter to record? Our data indicates that severity was also poorly judged. However, figures from three representative scenarios indicate that assessment of severity is more often associated with correct judgement of degree of contraindication than categorisation of reaction type.

We surveyed different HCW professions in proportions roughly similar to those who are responsible for ADR entry into the EHR, although in our state-wide EHR there is a higher proportion of nurses (58%) entering such data [13]. Our scenarios demonstrate a number of difficult areas with regard to HCW knowledge of ADR classification and risk of drug re-exposure. Whilst the “correct” answers may in some cases be debatable, with different sources providing different recommendations, we contend that these are evidence-based and current best practice [15]. Our aim was to examine clinician’s knowledge of common ADRs observed in practice, with the classification as ‘correct’ in the study reflective of the most common mechanistic reactions for the ADR, levels of severity and contraindications associated clinical scenarios presented. Gaps in knowledge include a small proportion clearly unaware of the seriousness of SJS, and hepatotoxicity and myelosuppression caused by azathioprine. There are disparities in reaction severity and level of contraindication; for example, angioedema is an absolute contraindication to ACE inhibitors (because re-exposure could cause fatal angioedema) and rhabdomyolysis, whilst severe, does not contraindicate cautious (low dose) re-exposure to the statin [20].

In this study, we provided the respondents the necessary diagnostic information to be able to categorise the reactions. In clinical practice, the classification and assessment of an ADR is dependent on the level of information provided within the context of a current ADR or a previous reaction. Expert assessment may be necessary in some cases to determine reaction mechanism. Proof of an allergic mechanism may be provided by allergy testing (specific IgE test, intradermal test, patch test or drug challenge) but this is only available and appropriate for a minority of drugs and reaction types. At the time of patient registration and entry of ADR information into the EHR, such information is seldom available.

This data leads us to argue that the categorisation of ADRs as allergy or intolerance, which is obligatory in some EHRs, is unlikely to be useful. Since allergy is not inherently or necessarily more dangerous than intolerance and, since we have demonstrated, HCW of varying educational backgrounds have difficulty making this distinction, we suggest that this should not be a structural requirement of the ADR module of the EHR. We suggest that the drug, the reaction type and reaction severity (using objective measures wherever possible) should be recorded. Mechanism and level of risk are not always obvious (for example, angioedema with an ACE-inhibitor is intolerance not allergy; the reaction may be moderate, but contraindication is absolute) and need not be specified at the time of ADR entry. The level of risk and therefore the degree of contraindication vary with drug and reaction type, as well as presence of cofactors and the passage of time. Concepts of risk and cross-reaction risks within and between drug families may change over time. Ideally the EHR would provide decision support by generating patient-customised, drug-specific risk level information by reference to a database. In the absence of this, an alert should be generated, and the prescribing clinician can judge the level of risk with reference to the detailed information on the index reaction and decide to override if perceived benefits of the medication outweigh the risks.

## Conclusion

In conclusion, this study of almost 400 Australian HCW has shown suboptimal understanding of classification of ADRs with regard to the type, severity and level of contraindication of future drug exposure. With increased utilisation of EHRs globally, it is imperative that alerting strategies be developed to strictly avoid re-exposure of patients to drugs which carry an appreciable risk of inducing a dangerous reaction, whilst minimising the avoidance of drugs which are of minimal risk or allowing the use of low-risk drugs where the benefits may be significant. The ultimate goal being to promote safe use of medications and improve patient outcomes. The EHR user interface for ADR documentation should not require clinicians to categorise reaction mechanisms but capture maximum information to allow future safe prescribing decisions, followed by referral for further diagnostic workup if allergy is actually suspected.

What is already known about this topic?

- The correct classification of an adverse drug reaction (ADR) as allergy (immunological) or intolerance (non-immunological) has important clinical implications, especially with regard to future exposure or avoidance of the drug.
- Mislabelling of a drug allergy in patient records may result in unnecessary avoidance of an effective drug and has been associated with poor health outcomes for patients.

What does this article add to our knowledge?

- Health professionals have suboptimal understanding of classification of ADRs with regard to the type, severity and level of contraindication of future drug exposure.
- ADR documentation in EHR should not require clinicians to categorise reaction mechanisms but capture maximum information to allow future safe prescribing decisions, followed by referral for further diagnostic workup if allergy is actually suspected.

## Appendix

- **Question 1** What type of reaction would you categorise this as? (*Allergy or intolerance*)
- **Question 2** How severe would you say this reaction is? (*Mild, moderate or severe*)
- **Question 3** In the absence of any further information what is your recommendation regarding future use of the implicated drug in this patient? (Absolutely contraindicated, relatively contraindicated (alternative drug preferred but could be tried if strongly indicated) or can be used in the future with additional caution)
- **Question 4** If 3 options were available for categorisation, what type of reaction would you categorise this as? (Allergy, intolerance or side effect)

**Table:**

	Clinical scenario	Correct answer (Q1–Q3)
1	David, 72, was prescribed carbamazepine for trigeminal neuralgia. After 20 days, he developed mucosal ulcerations, generalized skin blistering and eye inflammation consistent with Stevens–Johnson syndrome	
2	Maggie, 31, took pantoprazole for gastroesophageal reflux disease. She experienced mild headache after the second dose, which spontaneously resolved after cessation of the medication	
3	Florence, 88, has been taking pravastatin for dyslipidaemia for almost 15 years. She presented with muscle weakness and pain. She has a very high serum creatinine kinase (CK) level and rhabdomyolysis is diagnosed	
4	Lucas, 43, developed low red and white blood cell counts and hepatitis following treatment with azathioprine for systemic lupus erythematosus (SLE)	
5	Mario, 71, presented with an episode of gross swelling of the lower face. He had a similar milder swelling of the lip a month ago. 3 months ago he commenced perindopril for hypertension	
6	Sandra, 27, has a history of several transient episodes of “hives”, attributed to eating too much fruit. She takes aspirin 600 mg and suffers from florid urticaria and mild facial swelling. Later she takes ibuprofen 400 mg and has a similar but milder reaction	
7	Diana, 42, is prescribed a beta-blocker for hypertension and presents to her GP a week later complaining of some wheezing and shortness of breath when playing basketball	
8	Leo, 45, developed itchy red hands and feet, wheezing and then collapsed within half an hour of the first dose of a course of oral Augmentin	
9	Mark, 56, was given morphine for back pain after a lumbar spine surgery. Within 24 h he developed pruritic macular lesions on the trunk and the limbs	
10	James, 22, was given a course of erythromycin for throat infection. He developed several episodes of abdominal cramps and loose stools during this course of antibiotics	

## Abbreviations

ACE: angiotensin-converting-enzyme
ADR: adverse drug reaction
EHR: electronic health records
GI: gastrointestinal
HCW: health-care workers
ICD-10: international classification of diseases, tenth revision
NSAID: non-steroidal anti-inflammatory drug
PPI: proton pump inhibitor
SD: standard deviation
SPSS: statistical package for the social sciences
SJS: Stevens–Johnson syndrome
